# In vitro activities of 18 antimicrobial agents against *Staphylococcus aureus *isolates from the Institut Pasteur of Madagascar

**DOI:** 10.1186/1476-0711-6-5

**Published:** 2007-05-23

**Authors:** Frédérique Randrianirina, Jean-Louis Soares, Elisoa Ratsima, Jean-François Carod, Patrice Combe, Pierre Grosjean, Vincent Richard, Antoine Talarmin

**Affiliations:** 1 Institut Pasteur de Madagascar, BP 1274, Antananarivo 101, Madagascar

## Abstract

**Background:**

*Staphylococcus aureus*, one of the most frequently isolated pathogens in both hospitals and the community, has been particularly efficient at developing resistance to antimicrobial agents. In developed countries, as methicillin-resistant *S. aureus *(MRSA) has prevailed and, furthermore, as *S. aureus *with reduced susceptibility to vancomycin has emerged, the therapeutic options for the treatment of *S. aureus *infections have become limited. In developing countries and especially African countries very little is known concerning the resistance of *S. aureus *to antibiotics. In Madagascar no data exist concerning this resistance.

**Objective:**

To update the current status of antibiotic resistance of *S. aureus *in Antananarivo, Madagascar.

**Methods:**

Clinical *S. aureus *isolates were collected from patients at the Institut Pasteur of Madagascar from January 2001 to December 2005. Susceptibility tests with 18 antibiotics were performed by the disk diffusion method.

**Results:**

Among a total of 574 isolates, 506 were from community-acquired infections and 68 from nosocomial infections. There was no significant difference in the methicillin resistance rate between community-acquired strains (33 of 506; 6.5%) and nosocomial strains (3 of 68, 4.4%). Many MRSA isolates were resistant to multiple classes of antibiotics. Resistance to tetracyclin, trimethoprim-sulfamethoxazole and erythromycin was more common. Among MRSA isolates resistance rates to rifampicin, fusidic acid, gentamicin and ciprofloxacin were lower than that observed with other drugs easily available in Madagascar. No isolates were resistant to glycopeptides.

**Conclusion:**

The rate of methicillin-resistant *S. aureus *is not different between community-acquired and nosocomial infections and is still rather low in Madagascar.

## Background

*Staphylococcus aureus *is an important cause of serious infections in both hospitals and the community. *S. aureus *has been found to be the most frequently isolated pathogen causing bloodstream infections, skin and soft tissue infections, and pneumonia [1-3]. Unfortunately this pathogen has been particularly efficient at developing resistance to antimicrobial agents. Since the first isolation of methicillin-resistant *S. aureus *(MRSA) in the United Kingdom in 1961 [4], increasing rates of methicillin resistance among *S. aureus *strains have been a cause for concern, especially in developed countries. In addition, MRSA has become resistant to multiple other antimicrobial agents. Until recently, vancomycin was believed to have retained activity against all strains of *S. aureus*; therefore, the spread of MRSA has led to increased vancomycin usage and hence increased selective pressure for the development of resistance [5]. In developing countries, since vancomycin is hardly available due to its cost, resistance to this drug is not yet a problem. Resistance to elder and/or cheaper antibiotics such as macrolide-lincosamide-streptogramin, rifampin, ciprofloxacin, fusidic acid and trimethoprim-sulfamethoxazole is more important.

Data concerning resistance of *S. aureus *to antibiotics in Madagascar are rare. A previous study was conducted in 1997–1998 by the Institut Pasteur of Madagascar (IPM) on 231 community-acquired strains [6]. In this study, no strain was resistant to methicillin. Another study concerned only 35 strains isolated from urinary tract infections from January 2004 and April 2006 [7]. Some of these strains are included in the present study. Only 2 strains (8%) were resistant to methicillin.

The aim of the present study was to make an update on the susceptibility of *S. aureus *isolates from the IPM to various drugs and therefore to improve the empirical approaches to the therapy of serious infections.

## Materials and methods

### Bacterial isolates

Clinical *S. aureus *isolates were collected from patients presenting at IPM for various bacteriological exams or from samples taken from hospitalized patients and sent at IPM, from January 2001 to December 2005. Only one isolate per patient was included in the study. Criteria for nosocomial infection were all infections developed in a patient after 48 hours of hospitalization. Strains were considered as community-acquired when isolated from patients that have not been hospitalized recently or from patients before 48 hours of hospitalization. Initial identification was based on colony morphology, gram staining, catalase and agglutination tests with Pastorex Staph (Biorad, Marne la Coquette, France). All isolates were immediately stored at -70°C.

### Antibiotic susceptibility testing

Susceptibility to antibiotics was assessed by the disk diffusion technique on Mueller-Hinton agar. An inoculum of 10^6 ^CFU/ml was prepared as recommended by the Antibiogram Committee of the French Microbiology Society (CASFM) [8]. After 24 h at 37°C, the zone of inhibition was measured. For susceptibility to oxacillin, an inoculum of 10^7 ^CFU/ml was prepared and the plate was incubated at 37°C for 24 hours on Mueller-Hinton agar + 2% NaCl. Antibiotic disks were obtained from Biorad, Marne la Coquette, France.

The following 18 antibiotics were tested: oxacillin, penicillin, erythromycin, lincomycin, pristinamycin, vancomycin, teicoplanin, ciprofloxacin, tetracycline, minocycline, trimethoprim-sulfamethoxazole, rifampicin, fusidic acid, gentamicin, kanamycin, tobramycin, chloramphenicol and fosfomycin. The breakpoints for resistance were those recommended by the CASFM [8]. *S. aureus *ATCC 25923 was used as control.

The resistance rate was calculated as the number of non-susceptible isolates divided by the total number of isolates. Multidrug resistance was defined as resistance to penicillin and oxacillin plus three or more of the following agents: erythromycin, lincomycin, rifampin, ciprofloxacin, gentamicin, tetracycline, and trimethoprim-sulfamethoxazole.

Comparison of resistance rate between nosocomial or community-acquired strains and between MRSA and MSSA was based on Chi square test of Pearson or exact test of Fisher according to the distribution; p significant level considered was p < 0.05.

## Results

A total of 574 isolates from 506(88.2%) community-acquired and 68 (11.8%) nosocomial infections, excluding consecutive samples from the same patient, were collected. Strains were isolated from 367 females and 207 males (mean age. 34.4 years old 95%CI [32.9–35.9], range 1 month – 90 years old, sex-ratio M/F: 0.56). Concerning the origin of the community-acquired isolates, 212 (41.9%) were from genital tract infections, 177 (36.0%) from pus, 97 (19.2%) from urinary tract infections and 20 (4.0%) were from the respiratory tract infections. For nosocomial strains, most (38) were isolated from surgical wounds (55.2%), 15 from cutaneous pus (22.4%) and 15 from hemoculture (22.4%).

Overall, the prevalence of MRSA was 6.5% (33 of 506 isolates) for community-acquired strains, and 4.4% (3 of 68) for nosocomial infections (p = 0.5). Rates of resistance of methicillin-sensitive *S. aureus *(MSSA) and MRSA to the other antibiotics tested are shown in Table 1. Eight (22.2%) MRSA isolates were multidrug resistant (Table 2).

**Table 1:** Antibiotic resistance profiles of 538 MSSA and 36 MRSA isolates from patients presenting at the Pasteur Institute of Madagascar, as determined by disk diffusion.

	**MSSA**			**MRSA**			
	Number of isolates that were			Number of isolates that were			P
	S	I or R	Resistance rate (%)	S	I or R	Resistance rate (%)	
Penicillin	66	472	87.7	0	36	100	0.11
Erythromycin	469	69	12.8	24	12	33.3	<0.01
Lincomycin	509	29	5.4	29	7	19.4	<0.01
Pristinamycin	538	0	0.0	34	2	2.0	-
Kanamycin	505	33	6.13	26	10	27.8	<0.01
Tobramycin	521	17	3.2	27	9	25.0	<0.01
Gentamicin	532	6	1.12	32	4	11.1	<0.01
Ciprofloxacin	509	29	5.4	31	5	13.9	0.03
Tetracyclin	244	294	54.6	9	27	75.0	0.01
Minocyclin	466	72	13.4	26	10	27.8	0.01
Trimethoprim-sulfamethoxazole	458	80	14.9	22	14	38.9	<0.01
Rifampicin	517	21	3.9	31	5	13.9	0.01
Fusidic acid	493	45	8.4	29	7	19.4	0.02
Chloramphenicol	487	51	9.5	28	8	22.2	0.01
Fosfomycin	530	8	1.5	35	1	2.8	NS
Vancomycin	538	0	0.0	36	0	0.0	-
Teicoplanin	538	0	0.0	36	0	0.0	-

**Table 2:** Phenotypic resistance patterns of 8 multiresistant *S. aureus *isolates for eleven antibiotics

KAN	TOB	GEN	TCY	MNO	ERY	LIN	PRI	CIP	SXT	RIF
S	S	S	R	S	R	R	S	S	R	S
S	S	S	R	R	R	R	S	S	S	S
R	S	S	R	S	R	R	S	R	R	S
R	R	R	R	R	S	S	S	S	R	S
S	S	S	R	R	R	R	R	R	R	I
R	R	R	R	R	R	S	S	S	R	S
S	S	S	R	S	S	S	S	R	S	R
R	R	R	R	R	R	R	R	R	R	I

KAN: kanamycin, TOB: tobramycin, GEN: gentamycin, TCY: tetracycline, MNO:minocyclin, ERY: erythromycin, LIN: lincomycin, PRI: pristiniamycin, CIP: ciprofloxacin, SXT: Trimethoprim-sulfamethoxazole, RIF: rifampycin

Of the 74 MSSA and 7 MRSA isolates that were resistant to erythromycin, respectively 26 (35.1%) and 7 (100%) had the constitutive macrolide-lincosamide-streptogramin B (MLS_B_) resistance phenotype. Of the 7 MRSA, 2 (28.6%) had also the streptogramin A (MLSA) resistance phenotype.

Susceptibility for minocyclin was available for 239 (74.4%) tetracyclin resistant strains, only 82 (34.3%) were also resistant to minocyclin.

Of the 574 isolates, 47 (8.2%) were resistant to at least one of the three aminoglycosides tested. Resistance of the MRSA isolates to aminoglycosids was less than expected: 24.1% were resistant to kanamycin, 17.2% to tobramycin, and only 10.3% to gentamicin.

Resistance rate to minocyclin was significantly higher in community-acquired than in nosocomial infection. No significant difference was observed in other antibiotics (Table 3),

**Table 3:** Antibiotic resistance profiles of 506 Community acquired and 68 Nosocomial isolates, as determined by disk diffusion.

	**Community acquired**	**Nosocomial infection**	
	Resistance rate (%)	Resistance rate (%)	P
Penicillin	87.9	91.2	0.43
Oxacillin	6.5	4.4	0.50
Erythromycin	14.6	10.3	0.43
Lincomycin	6.1	7.3	0.51
Pristinamycin	0.4	0.0	0.56
Kanamycin	7.3	8.8	0.65
Tobramycin	4.5	4.4	0.96
Gentamicin	1.9	0	-
Ciprofloxacin	5.5	8.8	0.30
Tetracyclin	56.5	51.4	0.43
Minocyclin	15.8	2.9	<0.01
Trimethoprim sulfamethoxazole	16.8	13.2	0.44
Rifampicin	4.1	7.3	0.25
Fusidic acid	8.9	10.3	0.70
Chloramphenicol	10.1	11.7	0.66
Fosfomycin	1.6	1.5	0.94
Vancomycin	0	0	-
Teicoplanin	0	0	-

Multiresistant	1.58	0	-

There were no significant differences in the resistance rates to any antibiotic according to the site of infection, the age group or the year of isolation of the strains (data not shown).

## Discussion

Our study presented some limits. Indeed, probably not all strains in our study were responsible for infections since 241 strains were isolated from genital infections using swabs and these strains are often contaminants. Concerning the detection of methicillin resistance we have followed the guidelines of the French Committee for the Antibiogram which recommend to use oxacillin on MH + 2% NACL or cefoxitin on MH (which seems more reliable [9]), with incubation at 37°C [8]. The Clinical and Laboratory Standards Institute recommend incubation at 35°C [10]. Some recent articles show that indeed 35°C seems to be more reliable[9]. However we have tested in 2005 more than 100 strains using both oxacillin on MH + 2% NACL and cefoxitin and did not find any discrepancy. Using cefoxitin at 37°C, detection of methicillin resistance could be overestimated and the discrepancy using strains presenting a low level of resistance is not important [9]. In our study most of the strains are fully sensitive to nearly all antibiotics, therefore discrepancies are likely to be very rare and thus the rate of methicillin resistance presented in our study is likely to be very close to the reality.

The prevalence of MRSA has increased worldwide, as it is evident from many surveillance studies [2,3,11]. However, there are considerable differences between countries. The very highest rates of methicillin resistance among *S. aureus *isolates have been noted in developed countries and especially in Western Pacific Regions, both in community acquired and nosocomial infections [11]. Usually, the prevalence of MRSA is lower in developing countries as in Africa. In our study, resistance to methicillin (5.8%) is rather low. In other studies conducted in African hospitals, resistance to methicillin varied from 21 to 63% in South Africa, and from 21 to 31% in Cameroon, Nigeria, Kenya, Ivory-Coast and Ethiopia [12]. In contrast, in North Africa, it was less than 10% in Tunisia and Algeria [12]. Although rather low, the rate of resistance to methicillin has increased between 1997–1998 [6] and our study from 0 to 5.8%. In contrast, the overall resistance to other drugs has not increased.

In contrast to other studies, where resistance rates are higher in nosocomial infections [11], we did not find any significant difference in the rates of resistance to most of antibiotic between strains isolated from nosocomial or community-acquired infections. Of course the number of nosocomial strains in our study is rather low, since IPM is not located in a hospital and mostly realise tests for out-patients. Nevertheless it is very likely that this reflects the reality of the resistance rates in the hospitals of Antananarivo. Although curious, this may be explained by the fact that the antibiotics delivered at the hospitals are the same that those found at the chemist's and often, inpatients have to buy the medicines outside the hospital.

About one third of the MRSA isolates were resistant to multiple other antimicrobial agents, as previously noted in the literature. In general, elevated rates of multidrug resistance may emerge from diverse isolates of *S. aureus *under antimicrobial pressure or as a result of widespread person-to-person transmission of multidrug-resistant isolates [13]. The fact that only a few antibiotics are easily available in Madagascar, may explain why resistance to major drugs used in developed countries is not frequent in our study. Although resistance rates to other antibiotics is usually significantly higher than in MSSA, the rates are much lower than in most studies. In contrast, resistance rates to cheaper antibiotics such as tetracyclin and trimetoprim-sulfamethoxazole are higher than those observed in developed countries and are similar to that observed in African countries [11,12,14].

Apart from glycopeptides which remain the most efficient on MRSA strains, the rates of resistance of MRSA to pristinamycin (0.4%), rifampicin (10.3%), ciprofloxacin (10.3%) and gentamicin (10.3%), were much lower than those to other antibiotics. In Madagascar ciprofloxacin, trimethoprim-sulfamethoxazole, tetracyclin and erythromycin are the only widely available oral agents. Rifampicin is only used for tuberculosis treatment. Because of their low price and the low rate of resistance, ciprofloxacin or rifampicin in combination with gentamicin may be the more suitable treatment on MRSA strains.

In summary, the rate of methicillin resistance among *S. aureus *isolated at the Institut Pasteur de Madagascar, both from community-acquired and nosocomial infections, remains rather low compared to developed countries and many developing countries in Africa. This is important for Malagasy physicians since the preferred regimen for suspected staphylococcal infections are cloxacillin or amoxicillin plus clavulanic acid. However a nationwide survey should be undertaken to confirm these results and could be valuable for the selection of therapeutic alternatives.

## Competing interests

The author(s) declare that they have no competing interests.

## Authors' contributions

ER, JFC, PC and PG participated in the collection of strains and susceptibility testing. FR participated in the design of the study, the collection of strains and susceptibility testing. JLS and VR drafted the manuscript and performed the statistical analysis. AT conceived of the study, and participated in its design and coordination and drafted the manuscript. All authors read and approved the final manuscript.
